# Seroprevalence and risk factors of Chagas disease in a rural population of the Quixeré municipality, Ceará, Brazil

**DOI:** 10.1590/0037-8682-0247-2020

**Published:** 2021-03-08

**Authors:** Arduina Sofia Ortet de Barros Vasconcelos Fidalgo, Alanna Carla da Costa, Alberto Novaes Ramos, Luzia Kalyne Almeida Moreira Leal, Alice Maria Costa Martins, José Damião da Silva, Anderson Fuentes Ferreira, Francisca Mylena Melgaço Nunes, Francisco Aucélio Alves Marinho, Julieth Mesquita Lacerda, Maria de Fátima Oliveira

**Affiliations:** 1 Universidade Federal do Ceará, Programa de Pós-Graduação em Ciências Farmacêuticas, Fortaleza, CE, Brasil.; 2 Universidade Federal do Ceará, Faculdade de Medicina, Programa de Pós-Graduação em Saúde Pública, Fortaleza, CE, Brasil.; 3 Universidade Federal do Ceará, Departamento de Fisiologia e Farmacologia, Fortaleza, CE, Brasil.

**Keywords:** Chagas Disease, Trypanosoma cruzi, Seroepidemiologic studies, Epidemiology

## Abstract

**INTRODUCTION:**

This study estimated the seroprevalence and risk factors of Chagas disease (CD) in a population of the Quixeré municipality, Ceará.

**METHODS:**

We conducted serological methods to detect the *Trypanosoma cruzi* infection. The other variables were evaluated by a standardized questionnaire.

**RESULTS:**

The estimated prevalence of CD was 3.7%. Male sex, age >40 years, being farmers, low education level, origin from rural areas, and being born in Quixeré were significantly associated with infection.

**CONCLUSION:**

CD persists in this rural population of Northeast Brazil. Poverty, low education, and limited information regarding CD are critical issues that need to be addressed.

The northeastern region of Brazil is considered the second most affected area by a wide geographical distribution of triatomines^1^ ^,^ ^2^. The state of Ceará has extensive rural areas of the Caatinga ecosystem with critical social conditions related to poverty. In these areas, precarious houses shelter various species of triatomine transmitters of *Trypanosoma cruzi* such as *Triatoma brasiliensis*, *T. pseudomaculata*, *Panstrongylus megistus*, *P. lutzi*, and *Rhodnius nasutus* ^3^ ^,^ ^4^.

A high prevalence of Chagas disease (CD) has been observed in the state of Ceará (14.8%) since the first studies performed by Alencar, particularly in the municipality of Limoeiro do Norte^5^. In this municipality, estimated seroprevalence rates of 2.6% (4/154) and 4.2% (34/812) were observed in 2011 and 2013, respectively^6^ ^,^ ^7^. This region presents a high risk of vectorial transmission due to the high rate of natural infection by *T. cruzi* (estimated in 7.9%) in triatomines^8^.

A study carried out in the same municipality of the present study from 2009 to 2015 accounted for a high number of captured insects and triatomine infection rates with the predominance of the *T. brasiliensis*, the most important vector in the transmission of CD in Ceará^8^.

Seroepidemiological studies in endemic areas are relevant strategies to obtain updated information to support surveillance, control, and prevention measures. The aim of this study was to estimate the seroprevalence of CD among the rural inhabitants of the municipality of Quixeré, state of Ceará, Northeast Brazil.

We conducted a cross-sectional study from January to April 2015 to estimate the seroprevalence of the *T. cruzi* infection in subjects aged >2 years living in the municipality of Quixeré, state of Ceara. With a total area of 613.578 km² and a population of approximately 22,008 inhabitants in 2018, it is located in the microregion of Baixo Jaguaribe, surrounded by the municipalities of Jaguaruana, Limoeiro do Norte, and Russas (Figure 1).

**FIGURE 1:**
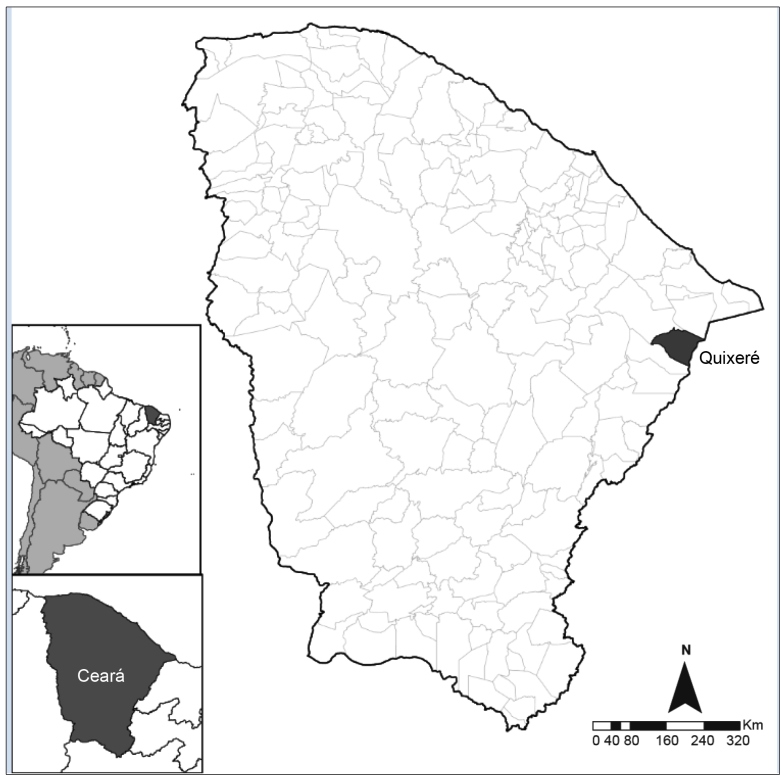
Map of Brazil, indicating the state of Ceará and the municipality of Quixeré.

A total of 4.5 mL of blood was collected from each participant. Anti-*T. cruzi* antibodies were evaluated using three conventional and standardized methods-enzyme-linked immunosorbent assay, indirect immunofluorescence, and indirect hemagglutination. Serological results were considered positive for the *T. cruzi* infection whenever at least two tests with different principles (one with high specificity and another with high sensitivity) were reactive. The risk factors were evaluated by a semi-structured questionnaire containing questions related to sociodemographic characteristics and individual risks.

The prevalence estimates and the respective 95% confidence intervals (95% CIs) were calculated. A descriptive analysis of the profile of the target population was conducted using the program Biostat 5.0. Fisher’s exact test was used considering a significance level of 0.05 and a 95% CI to determine the potential associations between positive results for CD and independent variables.

All the diagnosed CD cases were referred to Walter Cantídio University Hospital (Federal University of Ceara) for medical assistance. Further, cases with indications for antiparasitic treatment were referenced to the Pharmaceutical Assistance Service. This study was approved by the Research Ethics Committee of the Federal University of Ceara (n. 094708/2014), following the criteria of bioethics specified in Resolution 466/2012 of the National Health Council.

The characteristics of the included subjects (n=348) were as follows: females (234, 67.2%), age 21-40 years (126, 36.2%), family income of 1-2 minimum wages (193, 55.4%), low level of education (244, 70.1%), farming as occupation (164, 47.1%), and Quixeré as the place of birth (204, 58.6%).

A total of 242 (69.5%) subjects had a basic knowledge about triatomines. However, 206 (85,1%) participants had no knowledge about the eating habits of triatomines. Less than half (173, 49.7%) of the population were unaware of the seasons in which triatomines are more frequently identified in family homes or peridomiciliary areas. A total of 258 (74.1%) subjects knew that triatomines transmit diseases and 226 (64.9%) subjects knew that the insect transmits CD.

Most of the subjects (225, 64.6%) reported the presence of the vector inside family homes, but only 40 (11.5%) subjects had ever captured the insect and taken it to the municipal health surveillance services. The presence of domestic animals in residences or surrounding areas was reported by 295 (84.7%) subjects; dogs were the most common domestic animals (207, 59.5%).

On the basis of the serological analysis conducted in the municipality of Quixeré, the estimated prevalence of CD was 3.7% (13/348; 95% CI: 2.2%-6.3%). The rural locality of Boqueirão had the highest prevalence (5, 9.1%). The sociodemographic factors statistically associated with positive serology for CD were male sex, age >40 years, being farmers, low education level (incomplete basic education), family income of <2 minimum wages, and being born in rural areas of the municipality of Quixeré (Table 1).

**TABLE 1:** Statistical analysis of the main sociodemographic characteristics of the individuals with reactive serology for Chagas disease in Quixeré, Ceará, in 2015.

Characteristics	Positive	Negative	P-value
	N (%)	N (%)	
**Sex**			0.025*
Male	9 (7.9)	105 (92.1)	
Female	4 (1.7)	230 (98.3)	
**Age group**			0.010*
2 to 20 years	0 (0.0)	69 (100)	
21 to 40 years	0 (0.0)	126 (100)	
41 to 60 years	7 (7.1)	92 (92.9)	
61 to 80 years	5 (10.2)	44 (89.8)	
>80 years	1 (50.0)	2 (50.0)	
Unknown	0 (0.0)	2 (100)	
**Family income (monthly minimum wage)**			0.006*
<1	1 (0.7)	134 (99.3)	
1 to 2	11 (5,7)	182 (94.3)	
3 to 5	1 (7.1)	13 (92.9)	
Unknown	0 (0.0)	6 (100)	
**Level of education**			0.040*
≤8 years of formal education	13 (5.3)	231 (94.7)	
>8 years of formal education	0 (0.0)	104 (100)	
**Birthplace**			0.130*
Quixeré	11 (5.4)	193 (94.6)	
Other municipalities	2 (1.4)	142 (98.6)	
**Occupation**			0.075*
Farmer	6 (3.7)	158 (96.3)	
Retiree	5 (13.2)	33 (86.8)	
Student	0 (0.0)	52 (100)	
Homemaker	1 (1.8)	56 (98.2)	
Others	1 (2.7)	36 (97.3)	

*Fisher's exact test.

The statistical analysis of the biological risk factors of the individuals with positive serology for CD in Quixeré showed that most subjects knew that triatomine density is high in the rain season, making it the highest risk period for the transmission of CD.

Eleven (85%) of the 13 seropositive individuals reported having already lived in clay houses (Table 2), and four declared having donated blood sometime in their lives. There was no significant statistical difference between individuals with or without CD and the existence of comorbidities or private health plans. Comorbidities were reported by five individuals with positive serology for CD, two of whom had systemic high blood pressure.

The present study confirms the existence of an endemic area for CD in the municipality of Quixeré, Ceará. This fact is known from the time the first studies were performed by Alencar in 1963, showing an estimated seroprevalence of 22.5%^9^. We showed that the seroprevalence rate of CD in Quixeré was 3.7% higher than that reported in other studies performed in the state of Ceará (3.1%^10^, 1.2%^11^, and 1.2%^12^) and lower than that reported in a study performed by Freitas et al. (2017) in the municipality of Limoeiro do Norte (4.2%, 34/812)^11^. Limoeiro do Norte is historically known as an endemic area for CD due to the high prevalence of CD over many decades.

**TABLE 2:** Statistical analysis of the biological risk conditions of the individuals with reactive serology for Chagas disease in Quixeré, Ceará, in 2015.

Characteristics	Positive	Negative	P-value
	N (%)	N (%)	
**Wattle and daub house**			0.470*
Yes	11 (4.7)	223 (95.3)	
No	2 (1.8)	112 (98.2)	
**Donated blood**			0.029*
Yes	4 (11.8)	30 (88.2)	
No	9 (2.9)	305 (97.1)	
**Received blood (transfusions)**			0.603*
Yes	0 (0.0)	11 (100)	
No	13 (3.9)	324 (96.1)	
**Miscarriage**			1.000*
Yes	0 (0.0)	44	
No	4 (2.5)	154 (97.5)	
Unknown	0 (0.0)	32 (100)	
**Can identify the triatomine bug**			0.201*
Yes	11(4.5)	231 (95.5)	
No	2 (1.9)	104 (98.1)	
Knows that bug feeds on blood	11 (5.3)	195 (94.7)	0.354*
Does not know	2 (1.4)	140 (98.6)	
**Knows how the bug reproduces**			0.740*
Eggs	2 (2.6)	76 (97.4)	
Does not know	11 (4.1)	259 (95.9)	
**Knows the season of the year when the bug is most frequently present**			0.032*
Rainy	7 (8.0)	80 (92.0)	
Dry	4 (4.5)	84 (95.5)	
Does not know	2 (1.2)	171 (98.8)	
**Knows it transmits disease**			0.753*
Yes	9 (3.5)	249 (96.5)	
No	4 (4.4)	86 (95.6)	
**What disease?**			1.000*
Chagas disease	9 (4.0)	217 (96.0)	
Does not know	4 (3.3)	118 (96.7)	
**Reported the bug’s presence in the house**			0.783*
Yes	9 (4.0)	216 (96.0)	
No	4 (3.3)	119 (96.7)	
**What to do on finding the bug inside the house?**			0.568*
Kill it	11 (5.2)	200 (94.8)	
Capture it and advise the health center	1 (2.5)	39 (97.5)	
Others	1 (1.0)	96 (99.0)	
**Has animals inside the house or surrounding area**			0.700*
Yes	12 (4.0)	283 (96.0)	
No	1 (1.9)	52 (98.1)	
**Has a pet dog**			0.254*
Yes	10 (4.8)	197 (95.2)	
No	3 (2.1)	138 (97.9)	

*Fisher's exact.

Critical conditions of social vulnerability in rural areas in the northeastern region of Brazil reinforce the need to strengthen actions for disease surveillance and control and to integrate human and social development actions. We emphasize the relevant need to promote health education actions for the population of Quixeré, including general aspects of CD and the ecological characteristics of the triatomines. The search for greater community engagement through more active action in surveillance activities is crucial^13^.

The northeastern Brazilian region has the highest endemic factors for CD and has become the dispersion epicenter of two native species of triatomines which are highly difficult to control^1^ ^,^ ^2^-*T. brasiliensis* and *T. pseudomaculata*. The decline of CD transmission by the vector insect in Ceará is attributed mainly to the efficiency of vector control actions. The results of this study suggest that the municipality of Quixeré does not present an active vectorial transmission, mainly due to the absence of acute infection in individuals under 40 years of age. Thus, the findings of this study indicate that the infection was probably acquired in the past and was, therefore, in the chronic phase. A study performed in Quixeré by our research group estimated an infection rate of triatomines by *T. cruzi* of 2.6% in 2,123 specimens analyzed from 2009 to 2015. These results show that the endemic region is at risk of vectorial transmission. Therefore, the Chagas Disease Control Program must be intensified to guarantee the sustainability of the surveillance and control actions^8^ ^,^ ^13^.

In addition to actions aimed at controlling the vector and potential risk of reservoirs (such as dogs), it is important to expand accessibility to the diagnosis and treatment (parasitological and clinical complications) of CD^13^ ^,^ ^14^. To achieve this relevant goal, it is strategic to strengthen the epidemiological surveillance of chronic cases^13^ ^,^ ^14^. The cases diagnosed in this study that received parasitological treatment with benznidazole were referred for clinical follow-up at the CD reference center in the state of Ceará. Considering that benznidazol is a potentially toxic medicine, the main task is to follow up the diagnosed cases to allow for a more efficient and secure therapy^14^ ^,^ ^15^.

In conclusion, we demonstrate that CD persists as a public health problem in this rural population in Northeast Brazil. Poverty, low education level, and limited information regarding CD are critical issues that need to be addressed.
